# Compact, Energy-Efficient, High-Speed Electro-Optic Microring Modulator Based on Graphene-TMD 2D Materials

**DOI:** 10.3390/nano16030167

**Published:** 2026-01-26

**Authors:** Jair A. de Carvalho, Daniel M. Neves, Vinicius V. Peruzzi, Anderson L. Sanches, Antonio Jurado-Navas, Thiago Raddo, Shyqyri Haxha, Jose C. Nascimento

**Affiliations:** 1 Teleinformatics Engineering Department, Federal University of Ceara, Fortaleza 60455-970, Brazil; 2 CTI Center for Information Technology Renato Archer, Campinas 13069-901, Brazil; marchesi.daniel.neves@googlemail.com (D.M.N.); vinicius-vono.peruzzi@cti.gov.br (V.V.P.); 3 Engineering, Modeling & Applied Sciences Center, Federal University of ABC, Santo Andre 09210-580, Brazil; 4 Telecommunication Research Institute, University of Malaga, 29010 Malaga, Spain; navas@ic.uma.es (A.J.-N.);; 5 Department of Electronic Engineering, Royal Holloway, University of London, London WC1B 5DN, UK; shyqyri.haxha@rhul.ac.uk

**Keywords:** graphene, transition metal dichalcogenides, microring, modulator, power consumption, bandwidth

## Abstract

The continued performance scaling of AI gigafactories requires the development of energy-efficient devices to meet the rapidly growing global demand for AI services. Emerging materials offer promising opportunities to reduce energy consumption in such systems. In this work, we propose an electro-optic microring modulator that exploits a graphene (Gr) and transition-metal dichalcogenide (TMD) interface for phase modulation of data-bit signals. The interface is configured as a capacitor composed of a top Gr layer and a bottom WSe_2_ layer, separated by a dielectric Al_2_O_3_ film. This multilayer stack is integrated onto a silicon (Si) waveguide such that the microring is partially covered, with coverage ratios varying from 10% to 100%. In the design with the lowest power consumption, the device operates at 26.3 GHz and requires an energy of 5.8 fJ/bit under 10% Gr-TMD coverage while occupying an area of only 20 μm^2^. Moreover, a modulation efficiency of V_*π*_L = 0.203 V·cm and an insertion loss of 6.7 dB are reported for the 10% coverage. The Gr-TMD-based microring modulator can be manufactured with standard fabrication techniques. This work introduces a compact microring modulator designed for dense system integration, supporting high-speed, energy-efficient data modulation and positioning it as a promising solution for sustainable AI gigafactories.

## 1. Introduction

The unprecedented demand for artificial intelligence (AI) services and the continuous training of novel large language models (LLMs) are requiring a global surge in the construction of specialized data centers, commonly referred to as AI gigafactories, which are engineered specifically for the serving of these LLMs. These large-scale facilities, designed to host extensive computing and data-processing infrastructure, are rapidly emerging as major contributors to global energy consumption [1]. This wave intensifies pressure on already sutured electricity systems. As AI workloads expand exponentially, the associated power requirements are becoming a critical challenge for sustainable digital infrastructure. Training a single state-of-the-art model can consume several gigawatt-hours of electricity. In order to mitigate this growing energy footprint, the development of new energy-efficient technologies and device architectures has become imperative to cope with the energy demand of AI gigafactories. Against this backdrop, reducing the energy consumption per bit of optical transceivers, interconnects, and switching become part of the first-order design objective for a more sustainable AI data center infrastructure.

Traditional silicon photonics technology for electro-optic modulators and optical transceivers has made remarkable progress over this century in terms of modulation bandwidth, optical losses, energy consumption, and modulation efficiency [2,3,4,5,6]. Nevertheless, next-generation solutions have to further overcome challenges for meeting requirements such as energy-per-bit efficiency and high modulation bandwidth, to name a few, enabling a more sustainable and viable path toward reducing the overall energy consumption of future AI gigafactories. Emerging material platforms, such as graphene, transition-metal dichalcogenides (TMDs), thin-film lithium niobate [7], barium titanate [8], lead zirconate titanate [9], and a combination of them [10,11,12,13] are redefining the landscape of electro-optic devices, offering unprecedented opportunities to achieve high-speed, low-energy, and highly heterogeneous integrated devices.

Electro-optic modulators can be designed and engineered based on various material platforms. For instance, devices engineered from graphene exhibit the potential to yield superior performance, enabling higher bandwidth and modulation depth while sustaining low power consumption levels [14]. While TMDs exhibit electro-optic characteristics comparable to those of graphene, they differ in their thermal transport behavior and in the nature of their ambipolar carrier response. Graphene, a monolayer of carbon atoms fully compatible with CMOS fabrication, offers exceptional thermal stability, electrically tunable optical conductivity, and ultra-high carrier mobility, becoming a promising material for high-speed electro-optic modulation. By integrating graphene with a TMD layer, a hybrid heterostructure can be realized that combines the superior carrier transport and electrostatic tunability of graphene with the strong excitonic effects and optical field confinement inherent to TMDs. This synergistic combination enables enhanced electro-optic modulation efficiency, offering a promising alternative to current solutions.

A ring resonator modulator (RRM) based on multilayer graphene (Gr) integration was proposed in [15]. In such an RRM, intensity and phase modulation are achieved by shifting the resonance wavelength to selectively enable or suppress optical transmission at the target wavelength. Consequently, the tunability of the RRM is determined by the magnitude of the induced resonance shift per unit of applied voltage. The effective in-plane permittivity of graphene can be dynamically tuned through an externally applied gate voltage, which modifies its chemical potential and thereby enables active control of the device [16]. This Gr-based RRM demonstrates key performance advantages, including a large resonance shift, broadened electro-optic modulation bandwidth, high extinction ratio, and better temperature tolerance [17]. The improved thermal control of graphene is a key feature to provide thermal stability in next-generation AI chips with high-power density, which is essential for the deployment of AI hardware at scale [18].

Normally, TMDs have the chemical formula MX_2_, where M denotes a transition metal and X represents a chalcogen element. For example, a monolayer of WS_2_ consists of a tungsten (W) atomic layer sandwiched between two sulfur (S) atomic layers, forming an S-W-S structure. These units stack vertically to form layered crystals held together by van der Waals interactions, which enable the isolation or synthesis of few-layer and multilayer TMDs using a variety of techniques, including mechanical exfoliation, chemical vapor deposition, and atomic layer deposition [19]. Furthermore, the electronic, optical, and chemical properties of TMDs are strongly dependent on both layer thickness and stacking configuration, offering exceptional tunability and design flexibility.

The physical properties of TMDs differ markedly from those of graphene. While graphene is a zero-bandgap semimetal with exceptionally high electrical conductivity, TMDs exhibit finite bandgaps typically ranging from 1 to 2 eV, together with lower intrinsic carrier mobility. For example, MoS_2_ and WS_2_ have nonzero bandgaps and comparatively modest charge mobilities, resulting in reduced intrinsic carrier concentrations and electrical conductivity [20]. Nevertheless, these characteristics can be engineered through external apparatus such as mechanical strain, which has been shown to narrow the bandgap and enhance carrier density and mobility [21], thus enabling the creation of tunable devices.

The ability to change the bandgap is a defining feature of TMDs, making them promising candidates not only for electro-optic modulators [11], but also for photodetectors [22], high on/off-ratio field-effect transistors [23], piezoelectric nanogenerators [24], and gas sensors [25]. Moreover, the heterogeneous integration of TMDs with graphene yields hybrid heterostructures that leverage the complementary properties of both materials, consequently resulting in enhanced overall device performance. For example, a SiN-Gr-WSe_2_ phase modulator has been demonstrated to outperform its silicon counterpart [12], owing to its hybrid architecture, which enables large phase shifts with minimal optical loss and reduced amplitude variation. This design effectively mitigates the inherent trade-off between modulation efficiency and insertion loss, underscoring the potential of Gr-TMD heterostructures for high-performance integrated devices.

**Figure 1:**
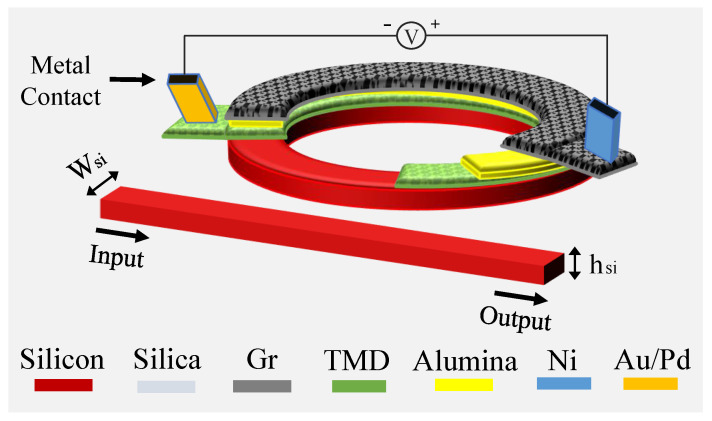
Schematic of a composite of the Si-Gr-WSe_2_ microring modulator. $W_{si}$ and $h_{si}$ are the width and height of both bus and ring waveguides.

Herein, a novel design for a Gr-TMD electro-optic microring modulator, engineered to achieve ultra-low energy per bit, large modulation bandwidth and high modulation efficiency, is introduced and investigated. The device comprises a silicon (Si) bus waveguide side-coupled to a Si ring waveguide, both embedded in silica (SiO_2_). On top of the ring, we integrate a capacitive hetero-stack of alumina (Al_2_O_3_) as the gate dielectric sandwiched between a top graphene sheet (Ni contact, positive electrode) and a bottom WSe_2_ layer (Pd/Au contact, negative electrode) to enable field-effect tuning of the complex effective index. The novel Si-Gr-WSe_2_ design strengthens optical confinement, due to the high refractive index of the Si platform, and overlaps with the 2D active region, thereby supporting further miniaturization, modifying the free spectral range (FSR), and amplifying the effective index perturbation for improved modulation efficiency. Electrode metallurgy is considered to lower contact resistance, enhancing the RC-limited electrical bandwidth. Figure 1 shows the design of the proposed microring modulator.

The performance evaluation of the proposed device is carried out considering the partial coverage of the ring by the Si-Gr-WSe_2_ stack from 10% to 100% in different discrete steps. The evaluation encompasses performance metrics such as modulation bandwidth, energy per bit, and a figure of merit combining bandwidth and energy, obtained via finite-element electromagnetic and circuit co-simulation using COMSOL Multiphysics 6.1 (build 252). Numerical results show a bandwidth increase from 26.3 GHz (10%) to 64.3 GHz (100%), with energy consumption rising from 5.8 fJ/bit (10%) to 23.4 fJ/bit (100%), and a concomitant improvement in modulation efficiency from 0.203 V·cm (10%) to 0.021 V·cm (100%). Notably, even at low Gr-WSe_2_ stack coverage, the modulator device sustains high-speed operation at femtojoule-level energy, with a 20 μm^2^ footprint supporting dense integration. This leads the way to the future realization of emerging applications with high integrability and scalability, such as 2D arrays of devices composed of hundreds of individual modulators for meeting the requirements of next-generation AI data-centers.

## 2. 2D Material Properties

This section presents the mathematical models that describe the properties of Gr and TMD, the relation between chemical potential, conductivity, and permittivity, and an approximation for the effective index expression.

### 2.1. Chemical Potential

When a gate voltage *V* is applied between the electrodes, it causes a change in chemical potential in both WSe_2_ and Gr. Then, following a simple capacitance model, the Gr-TMD carrier density is defined as [26]:

(1) $$\rho =\frac{\varepsilon_{0}\varepsilon_{Al}}{eh_{Al}}\left(V+V_{0}\right),$$

where $\varepsilon_{0}$ is the dielectric constant of the vacuum, $\varepsilon_{Al}$ is the relative permittivity of the Al_2_O_3_, $h_{Al}$ is the thickness of the Al_2_O_3_ layer, *e* is the charge of an electron and $V_{0}$ is the offset voltage caused by natural doping of Gr-TMD. Thus, the chemical potential (Fermi level) corresponding to the applied gate voltage is given by [27]:

(2) $$\mu_{c}=\hbar v_{F}\sqrt{\pi \rho },$$

where *ℏ* is the reduced Planck constant and $v_{F}$ is the Fermi velocity.

### 2.2. Graphene

The infinitesimally thin graphene monolayer is modeled as a two-sided surface with conductivity $\sigma_{g}\left(\omega ,\mu_{c},\Gamma ,T\right)$, where ω=0.8 eV is the light angular frequency, Γ=0.43 meV is the scattering rate of charged particles and *T* is the absolute temperature close to ambient air conditions. The conductivity of graphene, with its intra ($\sigma_{intra}$) and inter ($\sigma_{inter}$) band contributions, is obtained according to Kubo formula [28]:

(3) $$\begin{array}{cc} \sigma_{g}\left(\omega ,\mu_{c},\Gamma ,T\right)=\; & -\frac{ie^{2}}{\pi \hbar^{2}\left(\omega +i2\Gamma \right)}\int_{0}^{\infty }\epsilon \left(\frac{\partial f_{d}\left(\epsilon \right)}{\partial \epsilon }-\frac{\partial f_{d}\left(-\epsilon \right)}{\partial \epsilon }\right)d\epsilon \\ \; & -\frac{ie^{2}\left(\omega +i2\Gamma \right)}{\pi \hbar^{2}}\int_{0}^{\infty }\frac{f_{d}\left(-\epsilon \right)-f_{d}\left(\epsilon \right)}{{(\omega +i2\Gamma )\;}^{\;2}-4{\left(\epsilon /\hbar \right)}^{2}}\;d\epsilon \\ \; & =\sigma_{intra}+\sigma_{inter}^{\prime }+i\sigma_{inter}^{\prime \prime }, \end{array}$$

(4) $$\begin{array}{cc} \; & \sigma_{intra}=\sigma_{0}\frac{4\mu_{c}}{\pi }\frac{1}{\hbar (\tau_{1}-i\omega )},\\ \; & \sigma_{inter}^{\prime }=\sigma_{0}\left(1+\frac{1}{\pi }\arctan\frac{\hbar \omega -2\mu_{c}}{\hbar \tau_{2}}-\frac{1}{\pi }\arctan\frac{\hbar \omega +2\mu_{c}}{\hbar \tau_{2}}\right),\\ \; & \sigma_{inter}^{\prime \prime }=-\sigma_{0}\frac{1}{2\pi }\ln\frac{{\left(2\mu_{c}+\hbar \omega \right)}^{2}+\hbar^{2}\tau_{2}^{2}}{{\left(2\mu_{c}-\hbar \omega \right)}^{2}+\hbar^{2}\tau_{2}^{2}}, \end{array}$$

where $f_{d}\left(\epsilon \right)={\left(e^{\left(\epsilon -\mu_{c}\right)/K_{B}T}+1\right)}^{-1}$ is the Fermi-Dirac distribution with state energy ϵ, $K_{B}$ is the Boltzmann constant, $\sigma_{0}=60.8$ μS is the universal optical conductance, $\tau_{1}=1.2$ ps and $\tau_{2}=10$ fs are the inter-band and intra-band conductivity, respectively. Hence, the graphene in-plane permittivity can be computed as a function of $\sigma_{g}$ according to [29]:

(5) $$\varepsilon_{g}\left(\omega \right)=1+\frac{i\sigma_{g}}{\omega \varepsilon_{0}h_{g}}=1-\frac{Im(\sigma_{g})}{\omega \varepsilon_{0}h_{g}}+i\frac{Re(\sigma_{g})}{\omega \varepsilon_{0}h_{g}},$$

where $h_{g}$ is the thickness of the graphene layer.

### 2.3. TMD

The TMD monolayer follows a 2D conductive sheet model with optical conductivity given by [30]:

(6) $$\sigma_{t}\left(\epsilon \right)=-i\left(\varepsilon_{0}\epsilon /\hbar \right)\left(\varepsilon_{t}\left(\epsilon \right)-1\right),$$

where $\varepsilon_{t}\left(\epsilon \right)$ is the complex dielectric function in terms of a spectral range of photon energies {$\xi_{k}$: k∈N}, which is determined by a sum of Lorentzian oscillators:

(7) $$\varepsilon_{t}\left(\epsilon \right)=1+\sum_{k=1}^{K}\frac{f_{k}}{\xi_{k}^{2}-\epsilon^{2}-i\epsilon \nu_{k}},$$

where $f_{k}$ and $\nu_{k}$ are the strength and linewidth of the *k*th oscillator, and $\epsilon =\mu_{c}$ represents the Fermi energy. Also, it is possible to relate $\sigma_{t}$ and $\varepsilon_{t}$ in terms of $h_{t}$, the thickness of the TMD layer, as in [11]: $\sigma_{t}\left(\omega \right)=i\omega h_{t}\varepsilon_{0}\;\left(\varepsilon_{t}\left(\omega \right)-1\right)$.

### 2.4. Effective Index

The energy inside every material that composes the modulator structure is given by surface integration of the electric field for the TE mode, which is expressed as

(8) $$\gamma_{x}=∯E_{x}dS_{x},$$

where x∈{si,Al,air,t,g} is the subscript indicating the material. In this case, $E_{si}$, $E_{Al}$, $E_{air}$, $E_{t}$ and $E_{g}$ are the electric field inside the silicon, Al_2_O_3_, air, TMD and graphene regions; and $S_{si}$, $S_{Al}$, $S_{air}$, $S_{t}$ and $S_{g}$ are the corresponding surface areas. Furthermore, the permittivity of the entire modulator structure is calculated based on [17,31]:

(9) $$\begin{array}{cc} \varepsilon_{eff} & =\frac{\varepsilon_{si}\gamma_{si}+\varepsilon_{t}\gamma_{t}+\varepsilon_{Al}\gamma_{Al}+\varepsilon_{g}\gamma_{g}+\varepsilon_{air}\gamma_{air}}{\gamma_{total}}\\ \; & =\frac{12\gamma_{si}+10\gamma_{Al}+\gamma_{air}}{\gamma_{total}}+\frac{\varepsilon_{t}\gamma_{t}+\varepsilon_{g}\gamma_{g}}{\gamma_{total}}. \end{array}$$

where $\varepsilon_{si}$, $\varepsilon_{air}$ and $\varepsilon_{t}$ are the permittivity of silicon, air and TMD; $\gamma_{si}$, $\gamma_{Al}$, $\gamma_{air}$, $\gamma_{t}$ and $\gamma_{g}$ are the energy inside the silicon, Al_2_O_3_, air, TMD and graphene regions, and $\gamma_{total}$ is the sum energy of all regions. From (9), the effective mode index of the RRM can be derived as

(10) $$n_{eff}=\sqrt{\varepsilon_{eff}}\approx \sqrt{\frac{\psi }{\gamma_{total}}}\left(1+\frac{1}{2}\frac{\gamma_{t}\varepsilon_{t}+\gamma_{g}\varepsilon_{g}}{\psi }\right),$$

(11) $$\mathrm{with}\;\;\psi =12\gamma_{si}+10\gamma_{Al}+\gamma_{air}.$$

## 3. Principle of Operation for RRMs

**Figure 2:**
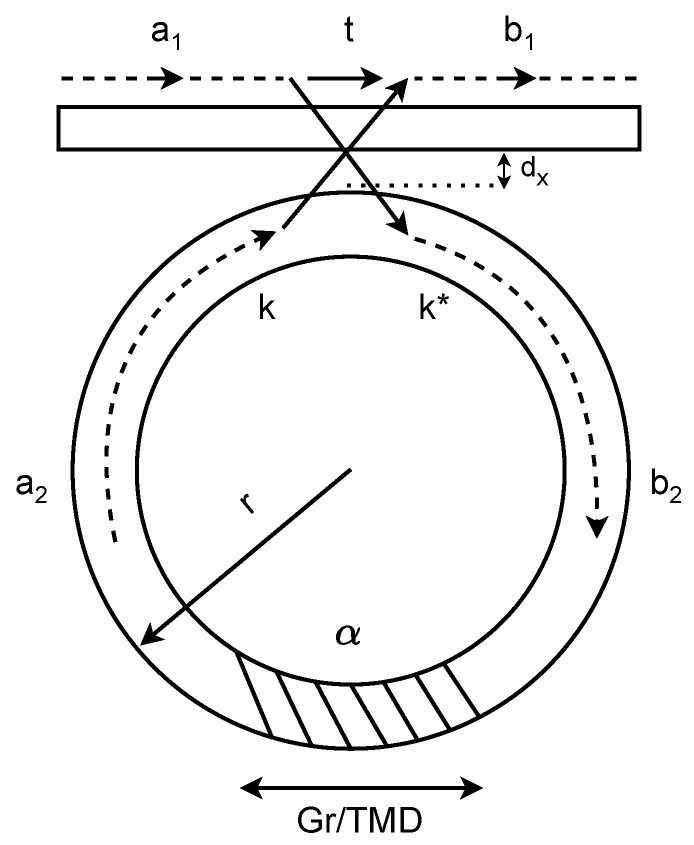
Ring modulator scheme showing input/output signals and key device parameters. The dashed area is the portion of the ring covered by the Gr-TMD interface.

Figure 2 illustrates the transmission path of lightwave in the waveguides and the process of transforming the input signal into the output. The input signal $a_{1}$ from the straight bus is coupled to the ring resonator, resulting in $b_{2}$, which after circling the ring, results in $a_{2}$. Then, the signal from the ring resonator is coupled to the straight bus, resulting in the output $b_{1}$. Given such process, the scattering matrix that defines the relation between those signals is expressed as [32]

(12) $$\left[\begin{array}{c} b_{1}\\ b_{2} \end{array}\right]=\left[\begin{array}{cc} t & k^{*}\\ -k & t \end{array}\right]\left[\begin{array}{c} a_{1}\\ a_{2} \end{array}\right],$$

(13) $$a_{2}=\alpha b_{2}e^{i\phi },$$

(14) $$\alpha =e^{-\left(\pi /\lambda \right)\;Im\left(n_{eff}\right)\;l},$$

where *t* and *k* represent the self-coupling and cross-coupling coefficients that satisfy $t^{2}+k^{2}=1$, α is the single-pass transmission through the ring, *r* is the ring radius with perimeter l=2πr, λ is the input wavelength and ϕ is the single-pass phase shift given by

(15) $$\phi =\left(2\pi /\lambda \right)\;Re\left(n_{eff}\right)\;l.$$

The expression that relates $b_{1}$ and $a_{1}$ is obtained from (12) in the following form:

(16) $$b_{1}=\frac{t-\alpha e^{-i\phi }}{1-\alpha te^{-i\phi }}a_{1}.$$

Hence, the transmission rate curve derived from (16) can be described as

(17) $$\tau \left(\phi \right)={\left|\frac{b_{1}}{a_{1}}\right|}^{2}=1-\frac{\left(1-\alpha^{2}\right)\left(1-t^{2}\right)}{{\left(1-\alpha t\right)}^{2}+4\alpha t\sin^{2}\left(\phi /2\right)}.$$

The transmission curve depends on the value of $n_{eff}$, which was computed using the finite element method (FEM) from the COMSOL Multiphysics software for a given wavelength and chemical potential. In order to obtain $n_{eff}$, both Gr and WSe_2_ monolayers are modeled by a boundary condition with surface current density given by J=σ·E, where *E* is the electric field vector and $\sigma =\sigma_{g}+\sigma_{t}$. Thus, α and ϕ can be determined through (14) and (15) for obtaining τ(ϕ) according to (17).

### Critical Coupling

Light modulation is achieved by active control of coupling, which can be performed through a swift tuning of α or *t*, causing the modulator to rapidly switch from critical coupling (α=t) to over (α>t) or under (α<t) coupling conditions. When the transmission curve is at resonance, ϕ=2πk with *k* integer, then (17) reduces to

(18) $$\tau \left(\phi \right)={\left|\frac{b_{1}}{a_{1}}\right|}^{2}=\frac{{\left(\alpha -|t|\right)}^{2}}{{\left(1-\alpha |t|\right)}^{2}}.$$

The result in (18) shows that τ(ϕ)=0 for α=t, meaning that the transmission at resonance is zero for critically coupled modulators. Following this principle, one can actively tune α from critical coupling (signal 0) to a certain value α>t with transmission level equal to 3dB (signal 1). This variation of α is caused by tuning the chemical potential of the Gr-TMD interface, which in turn alters the effective index, causing the change in α. Additionally, the cross-coupling coefficient *k* can be tuned by modifying the gap between ring and bus waveguides, $d_{x}$. Hence, by varying $d_{x}$, one can change *t* in order to achieve the critical coupling condition.

## 4. Performance Metrics

This section outlines the performance evaluation metrics for the proposed modulator. These metrics are the total bandwidth, given by the full width at half maximum (FWHM) and RC bandwidth, the switching energy between ON/OFF states, and the device total area (footprint), thus defining the modulator’s figure of merit. Moreover, the tuning efficiency (ratio between resonance shift and gate voltage variation) and modulation efficiency, given by the half-wave voltage length product (HWVLP), are also evaluated in the analysis.

### 4.1. Bandwidth Analysis

The modulation speed is determined by the electro-optic bandwidth, which is formed by the contributions of the RC circuit and FWHM. The equivalent resistance *R* and capacitance *C* of the Gr-Al_2_O_3_-WSe_2_ capacitor is required for computing the RC bandwidth as [33]:

(19) $$BW_{RC}\left(\mathrm{GHz}\right)=\frac{1}{2\pi RC},$$

(20) $$R=\frac{R_{c}^{t}+R_{c}^{g}+\left(R_{sh}^{t}+R_{sh}^{g}\right)G}{D},$$

(21) $$C=\frac{\varepsilon_{0}\varepsilon_{Al}}{h_{Al}}S,$$

where $R_{c}^{t}$ and $R_{c}^{g}$ are the TMD and graphene contact resistivity (Ω·m), $R_{sh}^{t}$ and $R_{sh}^{g}$ are the TMD and graphene sheet resistance (Ω/sq), *G* is the gap between capacitor and electrode, *D* is the length of the capacitor structure covering the ring circumference and *S* is the capacitor area. The FWHM with half maximum at −3dB can be computed as [34]:

(22) $$BW_{-3\mathrm{dB}}\left(\mathrm{nm}\right)=\frac{\left(1-t\alpha \right)\lambda_{res}^{2}}{\pi n_{g}L\sqrt{t\alpha }},$$

where $\lambda_{res}$ is the resonance wavelength, *L* is the round-trip length and $n_{g}$ is the group refractive index.

### 4.2. Energy Consumption Analysis

The modulator switches between ON and OFF states as the charge concentration is changed on both graphene and TMD layers. The increase in carrier density brings the modulator to the ON state (bit 1). The opposite behavior is expected when lowering the carrier density, thus bringing the modulator to the OFF state (bit 0). In order to switch between states, an energy cost per bit can be calculated according to [35]:

(23) $$E_{bit}=\frac{Q_{ON}^{2}-Q_{OFF}^{2}}{4C},$$

(24) $$Q_{ON}=\rho_{ON}eS=V_{ON}C,$$

(25) $$Q_{OFF}=\rho_{OFF}eS=V_{OFF}C,$$

where $Q_{ON}$ and $Q_{OFF}$ are the quantity of charge needed for bringing the modulator to ON and OFF states, $V_{ON}$ and $V_{OFF}$ are the gate voltages corresponding to ON and OFF states and $\rho_{ON}$ and $\rho_{OFF}$ are the carrier densities of ON and OFF states when $V+V_{0}$ equals $V_{ON}$ and $V_{OFF}$ in (1).

### 4.3. Figure of Merit

The modulator’s characterization can be described in terms of a figure of merit (FOM) that is defined by the performance metrics. The FOM definition follows the principle stating that the greater the FOM, the better the modulator performance. In order to evaluate data capacity (modulation bandwidth), power consumption (energy per bit) and footprint (area efficiency), the FOM is determined by [36]:

(26) $$FOM=\frac{BW_{total}}{E_{bit}\;Area},$$

where $E_{bit}$ is defined in (23) and Area represents the area of the whole device, considering the space occupied by both waveguides. The total bandwidth, composed of the contributions of optical and electrical bandwidths, is expressed as

(27) $$BW_{total}\left(\mathrm{GHz}\right)=\frac{1}{\sqrt{\frac{1}{{\left(BW_{-3\mathrm{dB}}\left(\mathrm{GHz}\right)\right)}^{2}}+\frac{1}{{\left(BW_{RC}\left(\mathrm{GHz}\right)\right)}^{2}}}}.$$

### 4.4. Modulation Efficiency

The tradeoff between power consumption and bandwidth is a key design factor in optical phase modulators. In addition to that, another tradeoff between modulation efficiency and footprint has to be considered. The smaller the variation in the effective index, the larger the device length for achieving a higher modulation effect, resulting in bigger insertion losses due to an increase in the propagation length. The HWVLP takes into account the round trip length, the FSR, the resonance shift, $\Delta \lambda_{res}$, and the corresponding gate voltage variation, $\Delta V_{\pi }$, for achieving an effective phase shift of π rad. Therefore, the HWVLP can be written as [15]:

(28) $$V_{\pi }L=\frac{FSR\;\Delta V_{\pi }\;L}{2\Delta \lambda_{res}}.$$

## 5. Simulation Results and Discussions

Computer simulations were conducted to assess the performance of the proposed modulator with eight different values of 2D material coverage: 10%, 15%, and 25% to 100% in steps of 15%. The dimensions of the resonator components are specified as ring radius r= 1.98 μm, Gr layer thickness $h_{g}=$ 1 nm, alumina layer thickness $h_{Al}=$ 7 nm, TMD layer thickness $h_{t}=$ 0.65 nm, and width and height of both core silicon waveguides $W_{si}=$ 400 nm and $h_{si}=$ 220 nm. Furthermore, the dielectric constants for silicon and alumina are $\varepsilon_{si}=12$ and $\varepsilon_{Al}=10$ [35], absolute temperature T=300 K and scattering rate Γ=0.43 meV.

The simulation parameters used for the device dimensions were chosen to satisfy a small footprint area requirement (at most 20 μm^2^) and, at the same time, provide a good trade-off between modulation efficiency and insertion loss. In fact, COMSOL simulations were performed in order to determine the value of $W_{si}$ (waveguides’ width) that provides the smallest value of $V_{\pi }$. In this case, the optimal value for the waveguide width was $W_{si}=400$ nm, which provides $V_{\pi }=0.469$ eV. Other values of $W_{si}$ and the corresponding $V_{\pi }$ are as follow: $\left(W_{si}\left(\mathrm{nm}\right),V_{\pi }\left(\mathrm{eV}\right)\right)=\left(350,0.477\right)$, (450,0.476) and (500,0.475). The modulation efficiency improves with smaller $V_{\pi }$; hence, the value of $W_{si}=400$ nm was chosen as the waveguides’ width. A small radius value (r<2 μm) was chosen to guarantee that the device footprint remains sufficiently small (close to 20 μm^2^), even though a narrower ring can lead to increased losses due to more sensitivity to fabrication defects. Lastly, the waveguide height, $h_{si}=220$ nm, and the dielectric thickness, $h_{Al}=7$ nm, were chosen according to [35], which were optimized in the context of RRM’s based on graphene.

### 5.1. Curves of Transmission for on/off States

The modulator setup demands fast switching between bits, driving the modulator from one logical state to another with a small variation of chemical potential. In order to achieve this, the OFF state was set for the modulator in the critical coupling condition, which drops the output transmission to zero. Then, by varying the chemical potential a few decimal units, the transmission at the desired wavelength is raised to unity and the resonant valley is shifted. This principle was described by Yariv [37], who states that small changes in *t* can control the transmission between unity and zero for α∼1. Table 1 shows the values of α in the critically coupled regime, which are near one for all coverage percentages, and the corresponding values of $d_{x}$ needed to achieve the critical coupling.

**Figure 3:**
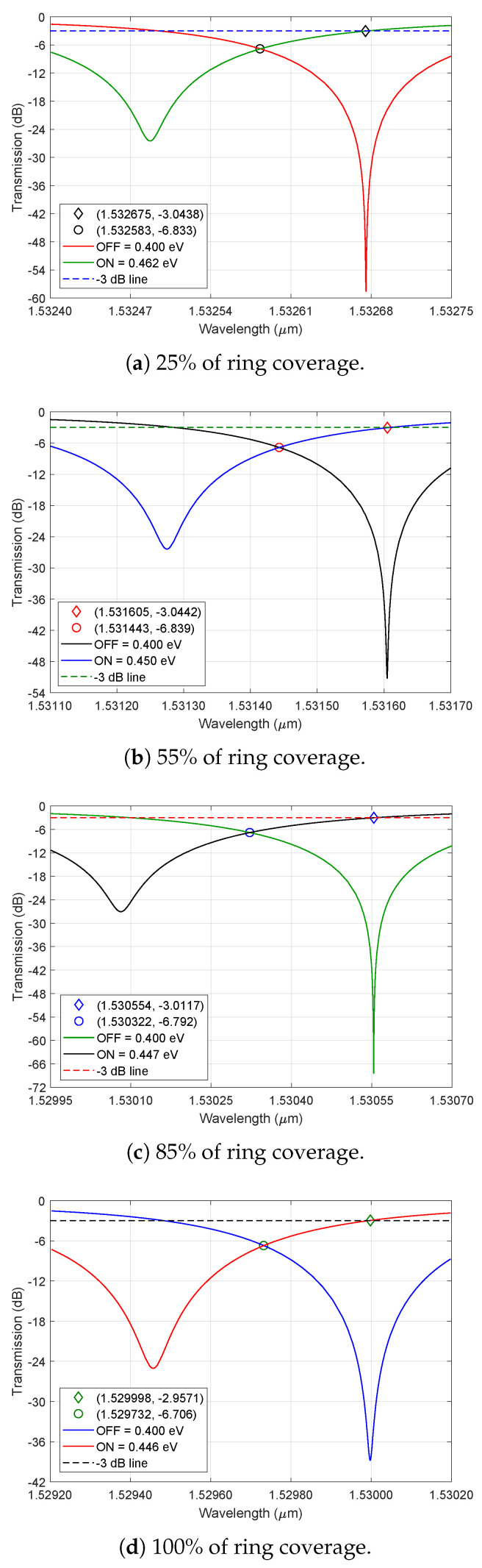
Microring modulator’s transmission curves for ON and OFF states with different amounts of ring coverage material.

In Figure 3a–d is shown the transmission curves (dB) as a function of the wavelength (μm) in four different cases: 25%, 55%, 85% and 100% coverage. The chemical potential for the OFF state was set to $\mu_{c}=0.4$ eV in all setups, while the ON potential was adjusted until the transmission reached a value near −3 dB (at the resonance wavelength of the OFF curve). The point that shows the value of $\lambda_{res}$ when the ON curve is at −3 dB (unity transmission) and the point where both curves meet is pointed out in the graphs. This indicates an insertion loss close to 6.8 dB in all cases, which is an acceptable loss. The extinction ratio (ER), that is, the difference of transmission between ON and OFF states, is displayed in Table 1. The chemical potentials obtained for the ON curves are $\mu_{c}=$ 0.496, 0.477, 0.462, 0.454, 0.450, 0.448, 0.447, and 0.446 eV, which correspond to 10%, …, 100% coverage. The range of resonance wavelengths is between 1.5300 μm and 1.5332 μm.

It is noteworthy that, as the Gr-TMD coverage decreases, the variation of chemical potential ($\Delta \mu_{c}$) required to switch between states grows since the resonator experiences an overall smaller shift in the mode’s path for smaller coverage. However, the higher quality factor due to smaller insertion loss in the cavity reduces the refractive index variation required for achieving the same resonance shift, which must be optimized in order to attain the best ring coverage configuration. While 2D materials can exhibit nonlinear optical responses such as saturable absorption at high power densities, the proposed modulator is designed to operate within the linear regime typical of silicon photonic interconnects. The sub-picosecond response time of graphene and the WSe_2_ interface ensures that the modulation depth and linearity remain stable at high-speed rates, effectively preventing signal distortion. At the calculated bandwidths and low energy-per-bit levels, the device’s performance is predominantly governed by the RC-limited electrical bandwidth and the optical quality factor of the microring resonator, ensuring reliable high-frequency operation for AI-driven data centers.

**Figure 4:**
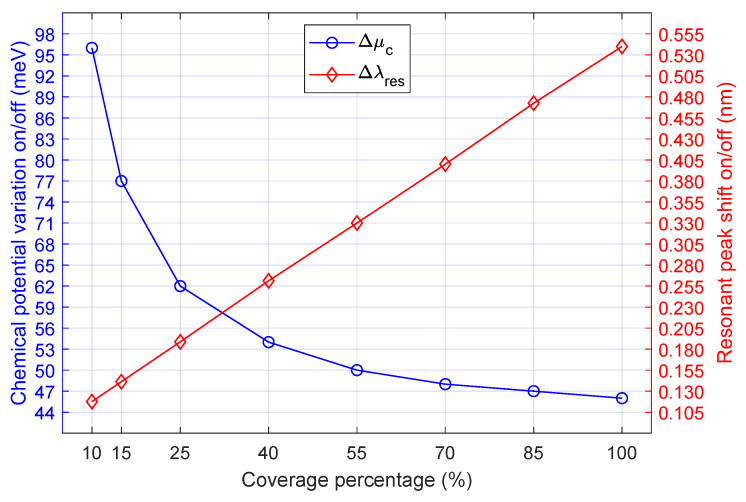
Chemical potential difference (meV) and resonance shift (nm) when switching between ON/OFF states versus Gr-TMD coverage.

**Figure 5:**
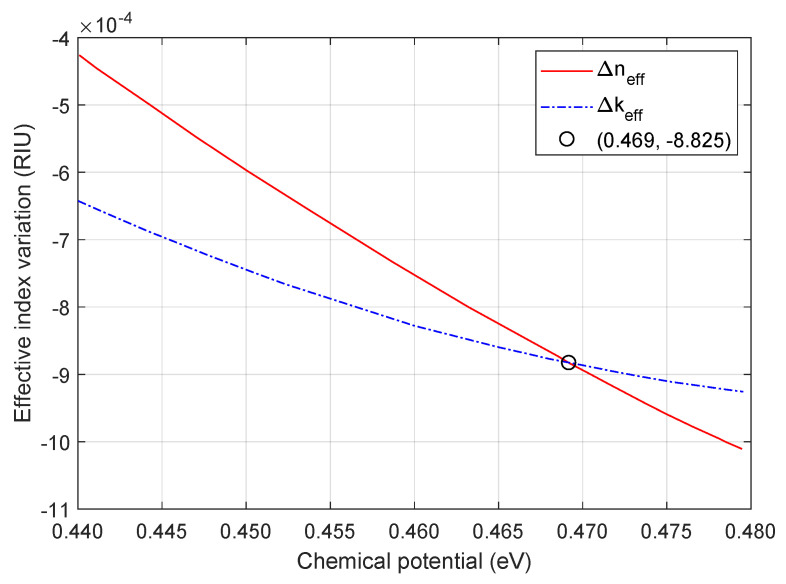
Variation of the effective index, measured in refractive index units (RIU), in terms of its real and imaginary parts versus chemical potential (eV).

Two key parameters, chemical potential variation and resonance shift, are represented in Figure 4, showing the values of $\Delta \mu_{c}$ and $\Delta \lambda_{res}$ for each coverage percentage. The amount of coverage material greatly influences both metrics since $\Delta \lambda_{res}$ increases approximately 0.075 nm for each increment of 15% and $\Delta \mu_{c}$ decreases exponentially as the coverage rises. On one hand, the resonance shift is almost tripled when the coverage varies from 25% ($\Delta \lambda_{res}=0.189$ nm) to 100% ($\Delta \lambda_{res}=0.540$ nm). On the other hand, the chemical potential variation is halved when the coverage increases from 10% ($\Delta \mu_{c}=96$ meV) to 70% ($\Delta \mu_{c}=48$ meV). The permittivity of Gr and WSe_2_ is tuned by the change of chemical potential, which influences the effective index as shown in (10). This means that $n_{eff}$ is complex, with $\Delta n_{eff}$ and $\Delta k_{eff}$ defined as the variation of its real and imaginary parts, respectively. $\Delta n_{eff}$ influences on the resonance shift, and $\Delta k_{eff}$ affects the transmission variation. The ratio $\Delta n_{eff}/\Delta k_{eff}$ is an important metric for evaluating the phase shift obtained when changing the modulator from the critical coupling to the over-coupling condition. As demonstrated by [12], if $\frac{\Delta n_{eff}}{\Delta k_{eff}}∼1$, then the modulator achieves a large phase shift (close to π) with low insertion loss and small amplitude modulation. Such a result was confirmed for our modulator for a specific value of chemical potential. This is demonstrated in Figure 5, which shows the curves for $\Delta n_{eff}$ and $\Delta k_{eff}$ and its meeting point at $\mu_{c}=$ 0.469 eV and $\Delta n_{eff}=\Delta k_{eff}=-8.83\times 10^{-4}$. The graph in Figure 5 is the same for all ring coverages since the proportion of variation of $n_{eff}$ depends only on the waveguide dimensions. This means that $V_{\pi }=$ 2.27 V and $\Delta V_{\pi }=V_{\pi }-V_{OFF}=$ 0.62 V.

**Figure 6:**
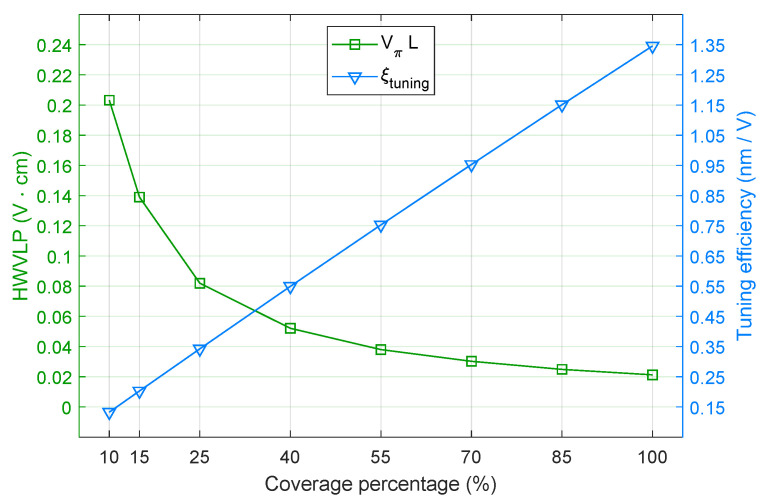
Modulation efficiency (V·cm) and tuning efficiency (nm/V) versus Gr-TMD coverage.

The next evaluation metrics are displayed in Figure 6, which shows the tuning efficiency, $\xi_{tuning}=\Delta \lambda_{res}/\Delta V$, and the HWVLP per coverage percentage. It is important to note that $\Delta V=V_{ON}-V_{OFF}$, and that the gate voltages are the values calculated from (2) for a certain $\mu_{c}$. For example, 10% of coverage gives $V_{ON}=$ 2.54 V and $V_{OFF}=$ 1.65 V, which results in ΔV= 0.89 V. This increase in gate voltage caused a resonance shift of $\Delta \lambda_{res}=$ 0.118 nm, corresponding to a tuning efficiency of 0.133 nm/V. The curve for $\xi_{tuning}$ is a straight line growing 0.202 nm/V per 15% coverage increase. Given this growth rate, $\xi_{tuning}=0.133+6\cdot 0.202=1.345$ nm/V for 100% coverage, which is about 10 times the tuning efficiency for 10%. This reveals that *m* times more coverage achieves *m* times more efficiency. The case of 25% ($\xi_{tuning}=0.342$ nm/V) compared to 100% shows a similar proportion: raising the coverage 4 times gives almost 4 times more efficiency.

The curve for $V_{\pi }L$ is a decreasing exponential that rapidly decays in the interval between 10% and 40%. In this case, the HWVLP drops from 0.203 to 0.052 V·cm, which is a reduction by almost a quarter. The second part of the curve, from 40% to 100%, decreases more slowly and reaches $V_{\pi }L=0.0201$ for the fully covered ring. In Table 1 are presented the values of FSR and the wavelength range between two resonances, which were used in the computation of (28). The FSR varies 0.028 nm per 15% coverage, with a mean of 44.834 nm. In addition to that, the round-trip length was set to L=2πr=12.441 μm.

The comparison between different TMD-based RRMs for phase modulation is shown in Table 2. Our device achieves superior modulation efficiency and tuning efficiency when compared to the other TMD platforms [11,12,13]. This enhanced performance includes an enlarged modulation bandwidth, albeit at the expense of increased insertion loss (IL). The lithium niobate modulator in [38] presents the lowest insertion loss (1.5 dB) with a modulation bandwidth of 30 GHz, although it provides a tuning efficiency of only 7 pm/V. The barium titanate (BaTiO_3_) modulator in [39] demonstrates the highest tuning efficiency (923 pm/V) and a large bandwidth of 65 GHz; nevertheless, it demands a high power consumption of 96 fJ/bit. Lastly, in [40], a modulator based on CuCrP_2_S_6_ (CCPS) provides a good modulation efficiency of 0.25 V cm and a bandwidth of 14.3 GHz, but its tuning efficiency is at most 8.3 pm/V. The modulation efficiency improves with higher ring coverage; however, adding more Gr-TMD increases device cost and power consumption, which has to be considered in the modulator design.

### 5.2. Power Consumption, Bandwidth, and FOM

**Figure 7:**
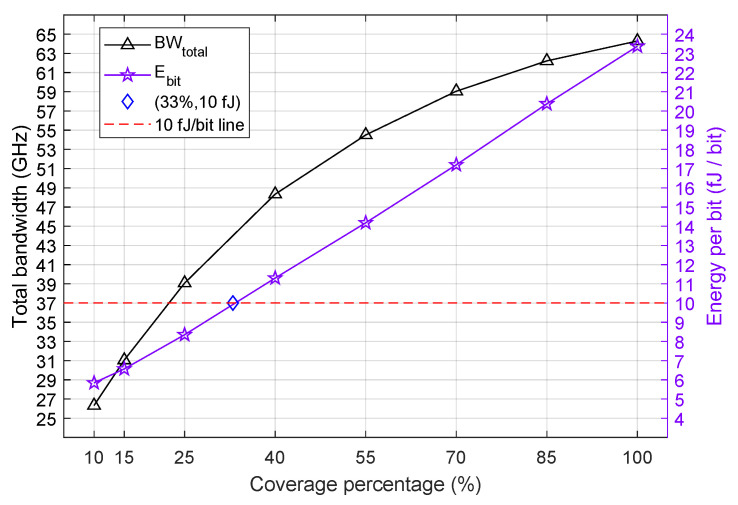
Modulation bandwidth (GHz) and power consumption (fJ/bit) versus percentage of 2D material coverage. The dashed line indicates the ideal energy consumption limit.

The compromise between modulation bandwidth and power consumption is depicted in Figure 7, which presents both metrics growing as the coverage percentage increases. This means that more bandwidth requires more energy to switch between logical states, which raises the cost of device operation for faster modulation. This increase in energy consumption is directly proportional to the capacitor’s capacitance, which is clearly seen when (24) and (25) are substituted in (23):

(29) $$E_{bit}=\frac{C}{4}\left(V_{ON}^{2}-V_{OFF}^{2}\right).$$

The energy cost per bit is also directly proportional to the difference of the squared gate voltages. This difference is smaller as the ring coverage grows since $\Delta \mu_{c}$ will decrease as well, as shown in Figure 4. Nevertheless, $E_{bit}$ will increase with more Gr-TMD coverage because the increment in *C* is more significant than the decrement in $V_{ON}^{2}-V_{OFF}^{2}$. The capacitor area *S* grows as the ring coverage is increased, which in turn increases *C* because of their relationship in (21). Consequently, the energy consumption grows linearly as the coverage percentage increases, approximately 3 fJ/bit for each 15% increment in ring coverage, as observed in Figure 7. Then, with such a proportion, 0.2 fJ/bit is added to the power cost for 1% more coverage. As proposed by D. Miller [41], the limit of switching energy for optical devices should be at most a few tens of fJ/bit, or ideally only 10 fJ/bit. In this case, our modulator spends 8.35 fJ/bit for 25% coverage and would reach 9.95 fJ/bit for 33% coverage if the rate of 0.2 fJ/bit per percent is kept. Therefore, the maximum coverage that respects the 10 fJ/bit limit is of 33%, as indicated by the energy consumption threshold line in Figure 7. Given a small tolerance, and with 40% coverage, the modulator will achieve a bandwidth of 48.33 GHz at the cost of 11.35 fJ/bit.

It is worth highlighting that, while the simulations were performed at a standard temperature of 300 K, the proposed Si-Gr-WSe_2_ architecture is designed to withstand the localized thermal effects common in high-power density AI environments. The integration of graphene provides enhanced thermal stability and efficient heat dissipation compared to traditional silicon-only resonators. Moreover, the ultra-low energy consumption (a few tens of fJ/bit) significantly reduces the risk of self-heating, ensuring that the device’s resonance remains stable even under the rigorous operational conditions of next-generation AI gigafactories.

The expressions for the electrical and optical bandwidths were defined in (19) and (22), respectively, and the modulation bandwidth was expressed in (27) in function of $BW_{RC}$ and $BW_{-3\mathrm{dB}}$. The contribution of the electrical bandwidth is the same for all coverages since the RC product is constant. The length of the capacitor around the ring is given by D=ζ·l, where 0<ζ<1 is the ring coverage, and the capacitor area is defined as $S=D\cdot W_{si}$. This means that the length contribution is canceled out in the RC product when multiplying (20) by (21). Hence, $BW_{RC}$ does not depend on the ring coverage percentage.

The total resistance *R* can be minimized by optimization of the sheet resistance, contact resistivity, and electrode capacitor gap. Several techniques can be employed to achieve high-quality electric contacts, such as metal-catalyzed etching for nickel-graphene contacts [42] and doping of WSe_2_, with tungsten oxyselenide (TOS) as dopant, for Pd/Au-WSe_2_ contacts [43]. These two techniques achieved a contact resistivity of $R_{c}^{g}=100$ Ω·μm for Gr [42] and of $R_{c}^{t}=321$ Ω·μm for WSe_2_ [43], both for monolayer devices. In order to ensure the practical relevance of the simulation results, the contact resistance values for graphene and WSe_2_ were adopted from established experimental reports utilizing advanced contact engineering techniques. Furthermore, the robustness of the design against fabrication fluctuations is evidenced by the consistent performance metrics obtained across a wide spectrum of coverage ratios (10% to 100%). This indicates that the modulator can tolerate minor alignment variations and interface non-idealities while maintaining its superior bandwidth and energy-per-bit characteristics, confirming its suitability for high-yield manufacturing in standard silicon photonics foundries.

These values of contact resistivity were used for computing (20), and the sheet resistances were set to $R_{sh}^{g}=200$Ω/sq [32] and $R_{sh}^{t}=1$ kΩ/sq [12]. The electrode capacitor gap was optimized to G=5 nm, according to [44]. This study employs high-resolution laser lithography, specifically laser direct writing (LDW), to reliably fabricate 5 nm nanogap electrodes. This type of technique is well known in the literature, with many established fabrication methods [45]. The electrical bandwidth obtained from those parameters is $BW_{RC}=73.67$ GHz. For 100% coverage, the equivalent resistance and capacitance are R=34.32 Ω and C=62.95 fF. If the coverage is reduced to ζ=1/m, then *R* will be multiplied by *m* and *C* divided by *m*, thus keeping $BW_{RC}$ constant. Optical bandwidth follows an opposite trend, increasing with coverage. This is true since t=α in the critical coupling regime, and for such a case, (22) can be written as a function of α:

(30) $$BW_{-3\mathrm{dB}}\left(\mathrm{nm}\right)=\frac{1-\alpha^{2}}{\pi \alpha }\;FSR,$$

(31) $$FSR=\frac{\lambda_{res}^{2}}{n_{g}L}.$$

The result in (30) reveals that the optical bandwidth is an increasing function with decreasing α because this is the same behavior of the function $f\left(\alpha \right)=\left(1-\alpha^{2}\right)/\alpha$. One can see in Table 1 an increase in bandwidth and a decrease in α as the ring’s coverage grows. The FSR, given by (31), is reduced for higher coverage as $\lambda_{res}$ becomes smaller. However, since its variation is very small, (30) is not significantly affected by the FSR and varies only on α. The curve for the total bandwidth is shown in Figure 7, as defined in (27). The curve grows faster for the first step of 15%, between 10% and 25% (12.7 GHz increase), and for every other step, the growth is continually smaller until it reaches a 2.1 GHz increase between 85% and 100%. This growth pattern is different from the one presented by $E_{bit}$, which grows uniformly as the ring coverage increases. This means, in terms of the FOM defined in (26), that the increase in coverage beyond 25% is not beneficial for the overall performance of the device since the ratio $BW_{total}/E_{bit}$ is progressively reduced.

**Figure 8:**
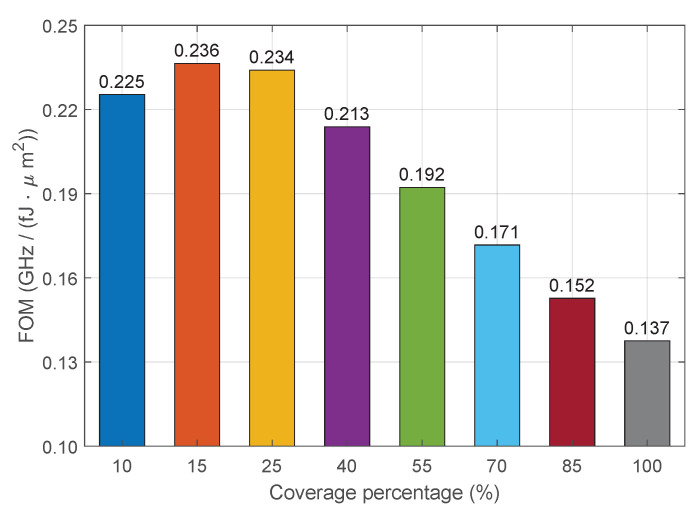
Modulator’s figure of merit (GHz/(fJ·μm^2^)) versus percentage of 2D material coverage.

The graph in Figure 8 shows the FOM (GHz/(fJ·μm^2^)) of our modulator for each coverage percentage and a total device’s area of 20 μm^2^. As expected, the greater values of FOM are found for 10%, 15%, and 25%, in which FOM>0.22. After this, the FOM decreases linearly in three steps of 0.021 between 25% and 70%, demonstrating that the FOM starts to degrade for ζ>0.25. This makes it clear that the increment in total bandwidth does not compensate for the increase in power consumption after the threshold of 25%. This happens because the FWHM grows almost linearly as the coverage increases, and the gain in GHz becomes proportionally low in relation to the higher values of bandwidth. This performance threshold for the FOM can be enlarged by increasing the RC-limited bandwidth with improvements in the sheet/contact resistances. Alternatively, the bit switching energy can be further reduced by choosing a dielectric material with smaller permittivity and/or greater thickness.

Hence, the analysis of the FOM indicates that a 25% coverage ratio represents the optimized configuration for the proposed modulator, yielding the highest balance between modulation bandwidth (39.1 GHz) and energy efficiency (8.35 fJ/bit). Beyond this threshold, the increase in power consumption disproportionately outweighs the gains in bandwidth, as confirmed by the degradation in FOM. This trade-off can be further optimized in future designs by tailoring the dielectric permittivity and thickness or by refining contact resistances to expand the efficient operational range of the device.

Finally, for the last two percentages, FOM<0.16 with a decrease of 0.019 from 70% to 85%, and of 0.015 from 85% to 100%. These values of FOM are comparable to RRM designs for phase modulation based solely on Gr. On one hand, the authors in [14] designed a ring modulator that achieves a bandwidth of $BW_{total}=34$ GHz, requires a switching energy of $E_{bit}=4.26$ fJ/bit, and occupies a footprint of 55 μm^2^. This results in FOM=0.145, which is close to our result obtained for 85% coverage (FOM=0.152). On the other hand, the work in [9] reported these performance metrics: $BW_{total}=88.45$ GHz, $E_{bit}=17.5$ fJ/bit, and footprint area of 22.1 μm^2^. This gives a FOM=0.229, which is comparable to our modulator for 10% coverage (FOM=0.225). The first modulator consumes less energy than our device; however, it requires more footprint area and provides a smaller bandwidth. The second modulator occupies a greater footprint in comparison to our device but provides a larger bandwidth at the cost of higher energy consumption.

Next, we carry out a sensitivity analysis considering some key performance metrics to understand how the device would behave under realistic manufacturing process variations. In evaluating how fabrication variations (especially ALD oxide thickness or dielectric constant) affect the switching energy, we consider two complementary and physically realistic limiting assumptions using the definition of $E_{bit}$ in (29). In a constant-V (driver-limited) scenario, the available voltage swing is set by the electronics (or breakdown/leakage constraints), so $V_{ON}$ and $V_{OFF}$ are approximately fixed as *C* varies; consequently, $E_{bit}\propto C$ and a thicker oxide (smaller *C*) reduces energy while a thinner oxide (larger *C*) increases energy. In a constant-Q (constant carrier swing/constant modulation strength) scenario, the device is biased to achieve the same target change in carrier density/chemical potential (and thus the same optical tuning), implying the required charge swing *Q* is approximately fixed; since Q=CV, a change in *C* is compensated by V∝1/C, and substituting into the same expression yields $E_{bit}=\frac{C}{4}\Delta V^{2}\propto 1/C$, i.e., a thicker oxide increases energy and a thinner oxide decreases energy. These two limits bracket practical operation depending on whether the modulator is constrained primarily by available drive voltage (constant-V) or by a required optical tuning target (constant-Q). Contacts dominate bandwidth robustness in 2D-material-based modulators: a large resistance penalty would reduce $BW_{total}$ by ∼30% (for ζ = 10%) and by ∼53% (for ζ = 40%). Waveguide-induced optical drift may cause a moderate bandwidth decrease. ALD oxide thickness shifts energy vs. RC trade-offs (thicker oxide increases energy while thinner oxide reduces energy).

### 5.3. Application-Oriented Design Choice

The modulator’s performance depends on the 2D material coverage percentage, and therefore, the applications of the proposed device are determined according to the Gr/TMD ring coverage. The analysis presented in this work shows that a design focused on low-power consumption would consider a ring coverage below 33%, which is the value that corresponds to the 10 fJ/bit ideal limit for switching energy. For such a case, the modulator’s performance was fully described in three different designs that suit the low-power requirement, which are: 10% design (5.8 fJ/bit), 15% design (6.6 fJ/bit), and 25% design (8.3 fJ/bit). Moreover, all the evaluated designs achieve a modulation bandwidth on the order of GHz, from 26.3 GHz (10%) to 64.3 GHz (100%), which favors high-speed operation in every coverage percentage above 10%. Finally, the device footprint of 20 μm^2^ ensures that the modulator can be integrated into high-density chips and arrays of multiple cascaded devices, making it suitable for applications that require high-density integration. It is worth mentioning that this device design is useful not only for AI data centers, but also for a broad range of emerging applications due to its large bandwidth and small form factor, such as free-space optical communications [46], passive optical networks [47], and 6G mobile systems [48], among others.

## 6. Fabrication of the Proposed Device

This section is dedicated to the discussion of fabrication methods of the Si-Gr-WSe_2_ RRM device and the challenges related to the manufacturing process, such as tolerance to fabrication errors and strategies for the mitigation of process variations.

### 6.1. Fabrication Feasibility

The proposed model of an RRM is composed of two different 2D material monolayers: graphene and WSe_2_. Certain semiconductor materials (e.g., germanium) are difficult to integrate with silicon; however, 2D materials can be easily integrated with silicon through transfer-based techniques [49]. Additionally, graphene and TMDs are compatible with the CMOS fabrication processes, which enabled the design of Si-SiN-integrated optical modulators based on graphene [16,17,27,32,35] and TMDs [11,13,49]. Recently, the paper in [12] reported an RRM integrated with silicon nitride waveguide based on a Gr-WSe_2_ 2D material platform. In [50], the authors demonstrated a TMD-based laser device fabricated from a silicon on insulator (SOI) wafer with MoS_2_-WSe_2_ heterostructure.

The aforementioned works show that 2D materials have been integrated in silicon-based photonic devices via feasible fabrication methods [51], such as chemical vapor deposition (CVD), atomic layer deposition (ALD), liquid–mechanical exfoliation, and conventional lithography techniques (e.g., ultraviolet (UV) and electron-beam lithography (EBL)). Therefore, the proposed modulator structure, Gr-Al_2_O_3_-WSe_2_ on Si waveguide, can be manufactured using these standard fabrication methods. In addition to that, the long-term stability and chemical integrity of the Gr-TMD interface are ensured by the 7 nm Al_2_O_3_ passivation layer and the total SiO_2_ encapsulation, which protect the 2D materials from environmental oxidation. Furthermore, graphene’s exceptional thermal conductivity and the device’s ultra-low energy consumption (5.8 fJ/bit) effectively minimize self-heating and localized thermal effects, ensuring robust operational integrity even under the high-power density conditions characteristic of next-generation AI gigafactories.

The proposed device uses a conventional SOI photonic platform (Si core waveguides with $W_{si}$ = 400 nm and $h_{si}$ = 220 nm) and a compact microring with r=1.98 μm (round-trip length L=12.441 μm), all compatible with standard lithography and etching used in silicon photonics. The active heterostack is a capacitor formed by Gr/Al_2_O_3_/WSe_2_ with $h_{g}$ = 1 nm, $h_{Al}$ = 7 nm, and $h_{t}$ = 0.65 nm, which is integrated on top of the ring over a controllable coverage ratio ζ from 10% to 100%. Metal contacts (Ni for graphene; Pd/Au for WSe_2_) are patterned by EBL/UV lithography. In particular, the required bus–ring coupling gap $d_{x}$ to reach critical coupling is in the range of 607–745 nm (Table 1), which is well within typical photonic lithography limits and significantly larger than sub-50-nm regimes.

### 6.2. Fabrication Process

We fabricate the SOI microring first and subsequently integrate the heterogeneous stack by wafer-level transfer of CVD graphene, lithographic definition of the active region, ALD deposition of a thin high-k gate dielectric (Al_2_O_3_ with appropriate nucleation/seeding), and finally transfer/patterning of WSe_2_ followed by CMOS-compatible metallization. This transfer-then-pattern approach is widely used in 2D-material integrated optoelectronics and allows the interaction length (coverage ζ) to be defined lithographically rather than being set by flake geometry [44,51,52,53]. To obtain the 2D material, one can grow a large-area graphene by CVD on a catalytic metal such as Cu, and then transfer it to the SOI wafer. Next, concerning WSe_2_, one can grow WSe_2_ by mechanically exfoliating WSe_2_ flakes and deterministically placing them on the ring region (which is fast, but has low scalability). As an alternative, one can CVD-grown WSe_2_ films on a growth substrate (e.g., sapphire/SiO_2_), and then transfer onto the SOI wafer, which is more scalable, but requires process control on uniformity and defect density.

### 6.3. Fabrication Challenges and Its Mitigation Strategies

Our proposed device is a Si bus waveguide side-coupled to a Si microring resonator embedded in SiO_2_, with an on-ring capacitive hetero-stack consisting of graphene/Al_2_O_3_/WSe_2_ (Ni contact to graphene; Pd/Au contact to WSe_2_), which enables the field-effect tuning of the complex effective index. In this section, we outline fabrication-relevant challenges for this 2D-material modulator and practical mitigation approaches.

Achieving repeatable, low-loss, wafer-scale placement of monolayer/few-layer 2D materials on pre-fabricated silicon photonic circuits without introducing contamination (polymer residues), wrinkles, tears, or non-uniform strain remains a challenge in wafer-scaling integration of graphene/TMD layers. As a mitigation strategy, one can use dry transfer (stamp-assisted pickup/placement) rather than fully wet PMMA transfer when possible, followed by vacuum anneal and gentle solvent cleans to suppress residue-induced optical loss and contact variability.

Moreover, conventional lithography and plasma etching can introduce defects, edge roughness, and unintentional doping, degrading mobility/sheet resistance and increasing optical absorption/scattering. This is particularly relevant because our electrical bandwidth model explicitly depends on sheet/contact resistances. In order to mitigate it, one can use low-damage etch chemistries and hard-mask strategies to reduce direct plasma exposure. Also useful is to add thin encapsulation/passivation after patterning to reduce ambient-induced drift (adsorbates) that can otherwise shift operating points over time.

In addition, conformal ALD growth can be non-ideal on pristine graphene due to limited nucleation, leading to pinholes, roughness, and trap density. In gated 2D photonics, dielectric quality might impact (a) capacitance density (energy/bit); (b) leakage (static power); (c) interface traps (required voltage swing and hysteresis). As an alternative, one should employ ALD seeding/functionalization prior to high-k deposition to ensure continuous films; run process splits to trade off capacitance density, leakage, and trap density; and apply post-deposition anneals within the thermal budget of the SOI backend to reduce traps and stabilize the dielectric/2D interface [11,51].

In 2D heterostructure devices, contact resistance variability is often the largest contributor to bandwidth spread because *R* sets $BW_{RC}$. Contact resistance depends strongly on interface cleanliness, contact geometry (edge vs. surface), metal choice, and anneal history. In order to attenuate them, one can use dedicated contact-region cleaning (localized descum/solvent cleans) prior to metallization; one should favor edge-contact-like geometries or contact-overlap layouts that reduce access resistance; and for WSe_2_, one should consider contact-region work-function engineering and mild doping/charge-transfer treatments compatible with the flow.

The device manufacturability becomes sensitive to lithography and gap-formation yield if the electrode gap is too small. For example, the feasibility of ≈5-nm nanogap electrodes has been experimentally demonstrated using super-resolution laser lithography, showing that such gaps can be obtained via advanced tooling and careful process control [44]. Lastly, regarding transfer and alignment challenges, the proposed device design leverages the maturity of CMOS-compatible processes like atomic layer deposition and electron-beam lithography to ensure high-quality interfaces. While process fluctuations can influence device performance, our methodology inherently addresses this by evaluating the modulator across a wide range of Gr-TMD coverage ratios, from 10% to 100%.

## 7. Conclusions

This paper proposed a novel electro-optic microring modulator based on a Gr-TMD heterointerface for efficient phase modulation. The intrinsic properties of graphene and WSe_2_, along with the operating principles of the proposed device, have been discussed in detail. COMSOL finite-element method simulations were conducted to obtain transmission spectra for eight different configurations of the microring with varying active-region coverage, ranging from 10% to 100% in increments of 15%. Furthermore, the analysis of the modulation performance has shown that minimal variations in the chemical potential are required for data-bit switching. The obtained resonance shift was sufficient to achieve high modulation efficiency while maintaining moderate insertion losses.

The figures of merit confirmed that the proposed modulator has a broadband response in the GHz range across all coverage material configurations. The device has low power consumption on the order of fJ/bit, which meets the stringent energy requirements of integrated systems. In addition, its compact footprint of 20 μm^2^ facilitates seamless integration with silicon photonics and microelectronic platforms. Thus, the Gr-TMD-based microring modulator achieved high modulation efficiency, high modulation bandwidth, and low energy consumption. These findings underscore the potential of Gr-TMD hybrids to enable power-efficient high-speed photonic devices essential for the continued performance scaling of next-generation AI gigafactories.

## Figures and Tables

**Table 1:** Key design parameters.

Coverage (%)	FWHM (GHz)	FSR (nm)	ER (dB)	$d_{x}$ (nm)	$\alpha_{\mathrm{critical}}$
10	28.20	44.910	52.82	745	0.9923
15	34.25	44.900	78.05	727	0.9907
25	46.10	44.880	55.53	700	0.9874
40	64.05	44.852	54.04	670	0.9826
55	81.01	44.824	48.19	649	0.9780
70	98.80	44.796	45.18	632	0.9733
85	116.2	44.769	65.41	617	0.9686
100	131.6	44.740	35.83	607	0.9646

**Table 2:** Performance Comparison of RRMs based on TMDs and Ferroelectric Materials.

References	Structure	$V_{\pi }L$ (V·cm)	$\xi_{\mathrm{tuning}}$ (pm/V)	BW (GHz)	IL (dB)
[11]	SiN-WS_2_	0.800	−	0.3	−
[12]	SiN-Gr-WSe_2_	0.612	1.54	14.9	5.2
[13]	SiN-MoS_2_	0.690	29.42	−	3.2
[38]	LiNbO_3_-SiO_2_	−	7.0	30	1.5
[39]	BTO-SiO_2_-Si	0.45	923	65	−
[40]	CCPS-SiO_2_-Si	0.25	8.3	14.3	−
this work-10%	Si-Gr-WSe_2_	0.203	133	26.3	6.7

All devices mentioned in Table 2 are compatible with CMOS fabrication methods.

## Data Availability

The data supporting the findings of this study are available in the article. Any other data may be obtained from the corresponding author upon reasonable request.

## References

[1] Google’s Environmental Report, 2024 and 2025. https://www.gstatic.com/gumdrop/sustainability/google-2024-environmental-report.pdf.

[2] Gardes F.Y., Reed G.T., Emerson N.G., Png C.E. (2005). A submicron depletion-type photonic modulator in silicon on insulator. Opt. Express.

[3] Green W.M.J., Rooks M.J., Sekaric L., Vlasov Y.A. (2007). Ultra-compact, low RF power, 10 Gb/s silicon Mach-Zehnder modulator. Opt. Express.

[4] Watts M.R., Zortman W.A., Trotter D.C., Young R.W., Lentine A.L. (2010). Low-voltage, compact, depletion mode, silicon Mach-Zehnder modulator. IEEE J. Sel. Top. Quantum Electron..

[5] Baba T., Akiyama S., Imai M., Usuki T. (2015). 25-Gb/s broadband silicon modulator with 0.31-V·cm *V*_π_*L* based on forward-biased PIN diodes embedded with passive equalizer. Opt. Express.

[6] Debnath K., Thomson D.J., Zhang W., Khokhar A.Z., Littlejohns C., Byers J., Mastronardi L., Husain M.K., Ibukuro K., Gardes F.Y. (2018). All-silicon carrier accumulation modulator based on a lateral metal-oxide-semiconductor capacitor. Photonics Res..

[7] Tiberi M., Montanaro A., Wen C., Zhang J., Balci O., Shinde S.M., Sharma S., Meersha A., Shekhar H., Muench J.E. (2025). Graphene Electro-Absorption Modulators for Energy-Efficient and High-Speed Optical Transceivers. arXiv.

[8] Neves D., Sanches A., Nobrega R., Mrabet H., Dayoub I., Ohno K., Haxha S., Glesk I., Jurado-Navas A., Raddo T. (2023). Beyond 5G Fronthaul Based on FSO Using Spread Spectrum Codes and Graphene Modulators. Sensors.

[9] Neves D., Nobrega R., Sanches A., Navas A.J., Glesk I., Haxha S., Raddo T. (2022). Power consumption analysis of an optical modulator based on different amounts of graphene. Opt. Contin..

[10] Carvalho J.A.D., Neves D.M., Nascimento J.C., Sanches A., Santos A.F.D., Cordette S.J., Haxha S., Jurado-Navas A., Raddo T. (2024). Ultra-Efficient Modulators Based on Chalcogenide and Graphene Materials for AI Hyperscalers. Proceedings of the 2024 SBFoton International Optics and Photonics Conference (SBFoton IOPC), Bahia, Brazil, 11–13 November 2024.

[11] Datta I., Chae S.H., Bhatt G.R., Tadayon M.A., Li B., Yu Y., Park C., Park J., Cao L., Basov D.N. (2020). Low-loss composite photonic platform based on 2D semiconductor monolayers. Nat. Photonics.

[12] Datta I., Molina A.G., Chae S.H., Zhou V., Hone J., Lipson M. (2024). 2D material platform for overcoming the amplitude–phase tradeoff in ring resonators. Optica.

[13] Chen H., Zhao Z., Zhang Z., Wang G., Li J., Shang Z., Zhang M., Guo K., Yang J., Yan P. (2022). Heterogeneous integrated phase modulator based on two-dimensional layered materials. Photonics Res..

[14] Chakraborty I., Debnath K., Dixit V. (2019). Low-energy high-speed graphene modulator for on-chip communication. OSA Contin..

[15] Zhang Z., Li J., Wang G., Shang Z., Chen H., Zhao Z., Zhang M., Liu F., Dong B., Guo K. (2022). Racetrack resonator based integrated phase shifters on silicon nitride platform. Infrared Phys. Tech..

[16] Zhou F., Hao R., Jin X.F., Zhang X.M., Li E.P. (2014). A Graphene-Enhanced Fiber-Optic Phase Modulator With Large Linear Dynamic Range. IEEE Photonics Technol. Lett..

[17] Du W., Li E.P., Hao R. (2014). Tunability Analysis of a Graphene-Embedded Ring Modulator. IEEE Photonics Technol. Lett..

[18] Kez D.A., Foley A.M., Wong F.W.M.H., Dolfi A., Srinivasan G. (2025). AI-driven cooling technologies for high-performance data centres: State-of-the-art review and future directions. Sustain. Energy Technol. Assess..

[19] Singh A.K., Kumar P., Late D.J., Kumar A., Patel S., Singh J. (2018). 2D layered transition metal dichalcogenides (MoS2): Synthesis, applications and theoretical aspects. Appl. Mater. Today.

[20] Lee E., Yoon Y.S., Kim D.J. (2018). Two-Dimensional Transition Metal Dichalcogenides and Metal Oxide Hybrids for Gas Sensing. ACS Sens..

[21] Choudhary M., Shital S., Yaakobovitz A., Niv A. (2020). Shear strain bandgap tuning of monolayer MoS2. Appl. Phys. Lett..

[22] Kakkar S., Majumdar A., Ahmed T., Parappurath A., Gill N., Watanabe K., Taniguchi T., Gosh A. (2022). High-Efficiency Infrared Sensing with Optically Excited Graphene-Transition Metal Dichalcogenide Heterostructures. Small.

[23] Zou T., Kim S., Reo Y., Heo S., Liu A., Noh Y. (2024). Electrical Properties of Electrochemically Exfoliated 2D Transition Metal Dichalcogenides Transistors for Complementary Metal-Oxide-Semiconductor Electronics. Adv. Electron. Mater..

[24] Zhang D., Yang Z., Li P., Pang M., Xue Q. (2019). Flexible self-powered high-performance ammonia sensor based on Au-decorated MoSe2 nanoflowers driven by single layer MoS2-flake piezoelectric nanogenerator. Nano Energy.

[25] Kim Y., Sohn I., Shin D., Yoo J., Lee S., Yoon H., Park J., Chung S., Kim H. (2024). Recent Advances in Functionalization and Hybridization of Two-Dimensional Transition Metal Dichalcogenide for Gas Sensor. Adv. Eng. Mater..

[26] Wu L., Liu H., Li J., Wang S., Qu S., Dong L. (2017). A 130 GHz Electro-Optic Ring Modulator with Double-Layer Graphene. Crystals.

[27] Liu M., Yin X., Avila E.U., Geng B., Zentgraf T., Ju L., Wang F., Zhang X. (2011). A graphene-based broadband optical modulator. Nature.

[28] Hanson G.W. (2008). Dyadic Green’s functions and guided surface waves for a surface conductivity model of graphene. J. Appl. Phys..

[29] Gosciniak J., Tan D.T.H. (2013). Theoretical investigation of graphene-based photonic modulators. Sci. Rep..

[30] Li Y., Chernikov A., Zhang X., Rigosi A., Hill H.M., Zande A.M., Chenet D.A., Shih E., Hone J. (2014). Measurement of the optical dielectric function of monolayer transition-metal dichalcogenides: MoS2, MoSe2, WS2, and WSe2. Phys. Rev. B.

[31] Hao R., Du W., Chen H., Jin X., Yang L., Li E. (2013). Ultra-compact optical modulator by graphene induced electro-refraction effect. Appl. Phys. Lett..

[32] Zhou F., Liang C. (2019). The absorption ring modulator based on few-layer graphene. J. Opt..

[33] Lee B.S., Kim B., Freitas A.P., Mohanty A., Zhu Y., Bhatt G.R., Hone J., Lipson M. (2020). High-performance integrated graphene electro-optic modulator at cryogenic temperature. Nanophotonics.

[34] Bogaerts W., Heyn P.D., Vaerenbergh T.V., Vos K.D., Selvaraja S.K., Claes T., Dumon P., Bienstman P., Thourhout D.V., Baets R. (2012). Silicon microring resonators. Laser Photonics Rev..

[35] Midrio M., Boscolo S., Moresco M., Romagnoli M., Angelis C.D., Locatelli A., Capobianco A.D. (2012). Graphene–assisted critically–coupled optical ring modulator. Opt. Express.

[36] Amin R., Ma Z., Maiti R., Khan S., Khurgin J.B., Dalir H., Sorger V.J. (2018). Attojoule-efficient graphene optical modulators. Appl. Opt..

[37] Yariv A. (2002). Critical coupling and its control in optical waveguide–ring resonator systems. IEEE Photonics Technol. Lett..

[38] Wang C., Zhang M., Stern B., Lipson M., Loncar M. (2018). Nanophotonic lithium niobate electro-optic modulators. Opt. Express.

[39] Abel S., Eltes F., Ortmann J.E., Messner A., Castera P., Wagner T., Urbonas D., Rosa A., Gutierrez A.M., Tulli D. (2019). Large Pockels effect in micro- and nanostructured barium titanate integrated on silicon. Nat. Mater..

[40] Dushaq G., Serunjogi S., Tamalampudi S.R., Rasras M. (2024). Electro-optic tuning in composite silicon photonics based on ferroionic 2D materials. Light Sci. Appl..

[41] Miller D.A. (2010). Optical interconnects to electronic chips. Appl. Opt..

[42] Leong W.S., Gong H., Thong J.T.L. (2014). Low-contact-resistance graphene devices with nickel-etched-graphene contacts. ACS Nano.

[43] Borah A., Nipane A., Choi M.S., Hone J., Teherani J.T. (2021). Low-Resistance p-Type Ohmic Contacts to Ultrathin WSe2 by Using a Monolayer Dopant. ACS Appl. Electron. Mater..

[44] Qin L., Huang Y., Xia F., Wang L., Ning J., Cheng H., Wang X., Zhang W., Peng Y., Liu Q. (2020). 5 nm Nanogap Electrodes and Arrays by Super-resolution Laser Lithography. Nano Lett..

[45] Luo S., Hoff B.H., Maier S.A., de Mello J.C. (2021). Scalable Fabrication of Metallic Nanogaps at the Sub-10 nm Level. Adv. Sci..

[46] Alvarez-Roa C., Alvarez-Roa M., Martin-Vega F.J., Castillo-Vazquez M., Raddo T., Jurado-Navas A. (2022). Performance Analysis of a Vertical FSO Link with Energy Harvesting Strategy. Sensors.

[47] Mbaret H., Bahloul F., Cherifi A., Raddo T., Karar A.S., Belghith A., Zayani H.M. (2024). Capacity Optimization of the Next-Generation Passive Optical Networks Based on Genetic Algorithm. Opt. Fiber Technol..

[48] Raddo T.R., Rommel S., Cimoli B., Vagionas C., Perez-Galacho D., Pikasis E., Grivas E., Ntontin K., Katsikis M., Kritharidis D. (2021). Transition technologies towards 6G networks. EURASIP J. Wirel. Commun. Netw..

[49] Yang S., Liu D.C., Tan Z.L., Liu K., Zhu Z.H., Qin S.Q. (2017). CMOS-Compatible WS2-Based All-Optical Modulator. ACS Photonics.

[50] Liu Y., Fang H., Rasmita A., Zhou Y., Li J., Yu T., Xiong Q., Zheludev N., Liu J., Gao W. (2019). Room temperature nanocavity laser with interlayer excitons in 2D heterostructures. Sci. Adv..

[51] Cheng Z., Cao R., Wei K., Yao Y., Liu X., Kang J., Dong J., Shi Z., Zhang H., Zhang X. (2021). 2D Materials Enabled Next-Generation Integrated Optoelectronics: From Fabrication to Applications. Adv. Sci..

[52] You J., Luo Y., Yang J., Zhang J., Yin K., Wei K., Zheng X., Jiang T. (2020). Hybrid/Integrated Silicon Photonics Based on 2D Materials in Optical Communication Nanosystems. Laser Photonics Rev..

[53] Wang B., Huang W., Chi L., Al-Hashimi M., Marks T.J., Facchetti A. (2018). High-k Gate Dielectrics for Emerging Flexible and Stretchable Electronics. Chem. Rev..

